# Reduction of a Multidrug-Resistant Pathogen and Associated Virulence Factors in a Burn Wound Infection Model: Further Understanding of the Effectiveness of a Hydroconductive Dressing

**Published:** 2014-12-10

**Authors:** Bonnie C. Carney, Rachel T. Ortiz, Rachael M. Bullock, Nicholas J. Prindeze, Lauren T. Moffatt, Martin C. Robson, Jeffrey W. Shupp

**Affiliations:** ^a^Firefighters’ Burn and Surgical Research Laboratory, MedStar Health Research Institute, Washington, DC; ^b^Department of Surgery, University of South Florida, Tampa; ^c^The Burn Center, Department of Surgery, MedStar Washington Hospital Center, Washington, DC

**Keywords:** infection, burn, wounds, dressings, virulence factors

## Abstract

**Objective:** Drawtex's ability to remove pathogens and associated virulence factors has been demonstrated in vitro. A model of burn wound infection was used to characterize the in vivo impact of this dressing on infection and wound healing. **Methods:** Paired burn wounds were created on the dorsum of Sprague Dawley rats and were inoculated with methicillin-resistant *Staphylococcus aureus* (MRSA). Animals were divided into 2 groups, half with wounds that received experimental dressing and the remaining half with control dressing-treated wounds. Dressings remained in place through 3, 6, 9, or 14 days after injury, and methicillin-resistant *S aureus* and virulence factors were quantified. Laser Doppler imaging was used to examine wound perfusion, and local host immune response was assessed through the quantification of mRNA expression. **Results:** By day 3, less methicillin-resistant *S aureus* was measured in wounds treated with experimental-dressing compared to control-dressing wounds. Quantities remained lower in the experimental group through day 14 (*P* < .001). More methicillin-resistant *S aureus* was quantified in the experimental dressing itself than in control dressing at all time points (*P* < .05). Experimental dressing-treated wounds contained less toxic shock syndrome toxin 1 and Panton-Valentine leukocidin than controls (*P* < .01) on days 6, 9, and 14. Induction of toll-like receptor 2, NOD-like receptor family, pyrin domain containing 3, and interleukin 6 was significantly lower in experimental-dressing treated wounds than in controls on days 6 and 9 (*P* < .05). **Conclusions:** The hydroconductive dressing provided a significant reduction in pathogen and virulence factors compared to a control dressing. As a result of clearance of virulence factors from the wound bed, a requisite alteration in host innate immune response was observed.

Infection is a significant cause of morbidity and mortality in the hospital setting, and it is an especially relevant factor in the care and treatment of burn-injured patients.^1^^-^3 The burn wound's microenvironment can provide a medium for the growth of pathogenic organisms and for their associated production of virulence factors.^3^ Proteinaceous virulence factors produced by many commonly encountered, wound-relevant pathogens can further impede normal wound healing by degrading existing viable tissue. There are many challenges to burn wound healing that may be exacerbated by infection, including burn depth progression, induction of septic shock, and the development of hypertrophic scar.^2^^-^4 Therefore, preventing invasive infection and reducing the load of virulence factors in the wound environment are critical to achieving rapid and complete wound healing and favorable outcomes in these patients.

Methicillin-resistant *Staphylococcus aureus* (MRSA), one of an increasing number of drug-resistant wound-relevant pathogens, produces virulence factors that have been found to induce shock and sepsis, and enhance bacterial survival. *S aureus* toxin serotypes include staphylococcal enterotoxin A through SEJ, and toxic shock syndrome toxin 1 (TSST-1).^5^ Many of the virulence factors produced by MRSA are categorized as superantigens, which are those exotoxins that have the ability to simultaneously bind both HLA-DR (or DQ) and the T-cell receptor, creating an immunological synapse that can produce inflammatory cytokines at pathologic levels both locally and systemically.^6^^-^10 Toxic shock syndrome toxin 1 is notable for its distinct properties ranging from toxicity induced lethal shock to environmental stability,^11^ and is among the most well-studied superantigens. Panton-Valentine leukocidin (PVL) is another virulence factor produced by *S aureus*, though it is not superantigenic. It is, however, a cytotoxin, and the presence of PVL is associated with increasingly virulent strains of MRSA.^12^ *S aureus* toxins have been shown to interact with nonimmune cells such as epithelial and endothelial cells as well.^13^^-^17

Many diverse topical agents and dressing products are available to clinicians treating patients with burn wounds. These agents range from ointments and creams to various dressing types, some products impregnated with antimicrobials, and some products without. Because of the prevalence of multidrug-resistant organisms and the risks associated with infection, antimicrobial agents are often preferentially selected over unimpregnated options. Unfortunately, many of the agents used, such as silver, are also known to have some amount of nontarget cytotoxicity that has the potential to impede the healing process.^18^^,^^19^ The obvious desired outcome for clinicians and patients is rapid and complete burn wound healing, free of complications. It is therefore essential to identify highly effective dressings that decrease infection while also imposing minimal toxicity to the host tissue.

Drawtex is a novel hydroconductive dressing product with a purported ability to remove significant amounts of tissue debris, bacteria, and exudate from wounds.^20^^-^22 It has been approved and indicated for use in a variety of wound types, including complex surgical wounds and burns, and leg, diabetic foot, and pressure ulcers. The dressing contains no antimicrobial chemical agents. A substantial ability (that significantly exceeded that of a comparable, routinely used control dressing) to take up multiple drug-resistant pathogens and associated virulence factors from various media has been previously demonstrated in vitro.^23^ Furthermore, in pilot studies, this experimental dressing was observed to reduce both bacteria and virulence factor levels in MRSA-infected burn wounds.^22^ While these studies indicate efficacy in decreasing pathogen and virulence factor levels, and therefore, the potential for significant positive outcomes in treating burn wound infections, a controlled in vivo study is needed to make the data translatable and potentially clinically relevant.

The present experiments were designed to further evaluate the efficacy of Drawtex (experimental dressing) as compared to a standard, non–antimicrobial-containing foam dressing (control dressing) in a well-powered in vivo model of burn wound infection. The capabilities of the dressing in removing pathogen and virulence factors from the wounds were examined by quantitative culture and ELISA (enzyme-linked immunosorbent assays). In addition, experimental and control-treated wounds were compared to determine if any changes in pathogen presence resulted in local biologic changes in the host wound tissue relevant to wound healing status. These comparisons were based on wound perfusion and host innate immune response.

## METHODS

### Animal model

The MedStar Health Research Institute Institutional Animal Care and Use Committee reviewed and approved this research, and animal care and handling was provided in accordance with standard operating procedures. In this study, 48 male Sprague Dawley rats (Harlan Labs, Frederick, Maryland) were received through our institute's animal facility, and a modified version of an established animal model^24^^,^^25^ was utilized. This model, in which animals are not known to become systemically ill or require resuscitative or other treatment measures, was selected for use in these experiments to allow for focused study of the local wound environment and local infection status. Briefly, animals were anesthetized and placed on a warming blanket, and their dorsum was shaved and depilated. Animal weights and temperatures were monitored throughout the course of the experiment, and no significant changes were seen in either metric in any animals. Baseline, preinjury biopsies were obtained from a site remote from the injury location. Paired burn wounds were created on the prepared skin, one on either side of the spine as described previously.^24^^,^^25^ Animals were subsequently grouped (n = 6) on the basis of the last day of experimentation (postburn day 3, 6, 9, or 14) and the type of dressing applied to their wounds (experimental or control).

On postburn day 1, wounds were inoculated with MRSA by placing a wound-sized gauze onto the burn wound. The gauze contained 200 μL of culture media containing approximately 1 × 10^8^ CFU of bacteria. The gauze was then sutured in place and covered with Mepitel One dressing (Mölnlycke Health Care US, LLC, Norcross, Georgia). On postburn day 2, the inoculation gauze was removed, wounds were biopsied and imaged, and a 3 cm × 3 cm piece of either control dressing or experimental Drawtex dressing was applied over each burn wound and sutured in place. The control dressing was selected for its similarities to the experimental dressing in terms of thickness and texture, and because it similarly does not contain silver or other antimicrobials. Both wounds on an individual animal were treated with the same dressing type (experimental or control). The entire area spanning the 2 wounds was then covered with Mepitel One dressing to prevent dressing removal. Animals were monitored with dressing kept in place through postburn day 3, 6, 9, or 14, at which time animals were weighed, anesthetized, and wounds were imaged with digital photography and laser Doppler imaging (LDI). Wound biopsies were collected using 2 mm punch biopsies. One third of the biopsies were placed in phosphate-buffered saline (PBS) with 0.5% Tween 20 (PBST; Sigma-Aldrich, St Louis, Missouri), while a second set was placed in AllProtect Reagent (Qiagen Inc, Valencia, California), and a third set was placed in sterile saline. The dressing was also biopsied with a 2 mm punch and samples were weighed, flash frozen in liquid nitrogen, and then stored at −80°C. Animals were necropsied on postburn day 3, 6, 9, or 14 and the entire wound area was collected and fixed in formalin for histological analysis.

### LDI capture and analysis

On the same postburn time points outlined earlier, LDI was used to assess wound perfusion. The technique provided a reliable and quantifiable means to assess perfusion in the present set of experiments. A Moor LDI-2 (Moor Instruments Ltd, Axminster, UK) was used to capture images for each wound. Perfusion units are determined based on the Doppler frequency shift of 2-mW helium-neon gas laser (632.8 nm) scattered by red blood cells in microcirculation.^26^^,^^27^ Images were taken using the same scan area dimensions at a constant distance from the wound surface. Using Moor LDI image processing software version 5.3 (Moor Instruments Ltd, Axminster, UK), flux images of each wound were analyzed for mean perfusion units within regions of interest.

### Quantitative cultures

The flash frozen dressing samples were homogenized using a TissueLyser LT (Qiagen Inc, Valencia, California), reconstituted with sterile PBS, and vortexed. Wound biopsies were weighed and immediately homogenized in sterile saline using a LabGen Homogenizer (Omni International, Kennesaw, Georgia). Both homogenates were serially diluted with 100 μL of each dilution plated on Mannitol salt agar plates (BD Biosciences, Franklin Lakes, New Jersey) selective for Staphylococcal species. After incubation at 37°C for 48 hours, yellow colonies (indicating coagulase positivity and presumptive pathogenic Staphylococcal species) were counted and CFU per gram (CFU/g) of dressing or skin was calculated.

### Virulence factor quantification

Enzyme-linked immunosorbent assays were used to quantify TSST-1 and PVL. Skin and dressing biopsies were collected, preserved, and processed as described earlier with homogenates reconstituted in PBST. Aliquots of 100 μL were added to wells of 96-well immunoassay plates (Nalge Nunc International, Rochester, New York) coated with 100 μL of a 1 mg/mL primary antibody raised to TSST-1 (Toxin Technology Inc, Sarasota, Florida) or PVL (Integrated Biotherapeutics, Gaithersburg, Maryland). Biopsy homogenates and a serial dilution of standard purified TSST-1 or PVL were treated in the same way. The plates containing samples and standard curve were then incubated at 37°C for 2 hours, washed with PBST, and 100 μL of secondary antibody diluted 1:300 in PBST was then added to each well. The plates were placed on a shaker and incubated at 37°C for 1 hour and then washed with PBST. One hundred microliter of 2,2′-Azino-bis(3-ethylbenzothiazoline-6-sulfonate) with 0.05 M phosphate citrate buffer (Sigma-Aldrich, St Louis, Missouri) and hydrogen peroxide was then aliquoted into each well. The plates were sealed and incubated at room temperature in the dark, and 100 μL of 0.5% of Sodium dodecyl sulphate in distilled water stopped the reaction. The plates were read at 405 nm in a VICTOR Multilabel Counter (PerkinElmer, Waltham, Massachusetts).

### Immunofluorescence

To visualize bacteria and virulence factor in wound biopsies, punch biopsies taken from areas of burn wounds were fixed in 10% formalin and embedded in paraffin. Paraffin blocks were sectioned at a thickness of 5 μm and were dried overnight. Slides were deparaffinized and rehydrated with PBS. Antigen retrieval was performed in Tris-EDTA buffer at 95°C to 100°C for 20 minutes. Slides were then cooled for 5 minutes in running cold water. Slides were washed in 0.025% Triton X-100 for 5 minutes × 2. Slides were blocked in 5% nonfat milk, 1% BSA, in PBS for 1 hour. After blocking, slides were incubated with mouse monoclonal anti–*S aureus* (Abcam, Cambridge, UK) or rabbit polyclonal anti-TSST-1 (Toxin technology, Sarasota, Florida) primary antibody diluted in PBS-0.05% Tween-20 at 4°C overnight. Negative control slides were incubated with PBS-Tween 20 only. After overnight incubation with primary antibody, slides were rinsed with 0.025% Triton-X-100 for 5 minutes × 2. Slides were then treated with goat anti-mouse-CY3 conjugated (Abcam, Cambridge, UK) or goat anti-rabbit-CY5 conjugated (Abcam, Cambridge, UK) secondary antibodies diluted in PBS-0.05% Tween-20 for 1 hour at room temperature. Slides were rinsed with PBS for 5 minutes 3 times and then counterstained with DAPI (Santa Cruz Biotechnology, Dallas, Texas) for 10 minutes. Slides were then viewed with a Zeiss Axioimager microscope and multichannel black and white camera equipped with fluorescence filters (Carl Zeiss, Oberkochen, Germany).

### Host gene expression analysis

A group of genes involved in the innate immune response were assayed for changes in transcript-level expression in the wounds using real-time reverse transcriptase polymerase chain reaction (RT-PCR). The genes assayed have ligand specificity to gram-positive bacteria^28^^,^^29^ or downstream relevance to the inflammatory response. After wound biopsies were removed from AllProtect preservative reagent, they were homogenized using the TissueLyser LT, and RNA was extracted using the RNEasy Fibrous Tissue Mini kit (Qiagen Inc., Valencia, CA). RNA sample quality, as indicated by 260/280 ratio, and concentration were obtained using a Nanodrop 2000c spectrophotometer (ThermoFisher, Waltham, Massachusetts). RNA samples were diluted to 1 ng/μL and assayed using the iScript One-Step RT-PCR Kit with SYBR green (Bio-Rad Laboratories, Irvine, California) with primers (Integrated DNA Technologies, Coralville, Iowa) and reverse transcriptase in 96 well plates according to the kit protocol. Glyceraldehyde 3-phosphate dehydrogenase (GAPDH) was used as a reference gene, and levels were quantified in all samples in parallel with target genes. Gene-specific primers were used for GAPDH and genes of interest and are detailed in Table 1. Primer sequences for toll-like receptor 2 (TLR2) were based on previous publications,^30^ while GAPDH, NOD-like receptor family, pyrin domain containing 3 (NLRP3), and interleukin 6 (IL6) primer sequences were designed using Primer3 software.^31^ Reactions were run in the Bio Rad CFX96 Real-Time PCR Detection System (Bio-Rad Laboratories, Irvine, California) and cycled according to the following: 50°C for 10 minutes, 95°C for 5 minutes, 95°C for 10 seconds, 54.5°C for 30 seconds (with repeating of steps 3–4 for 39 cycles), followed by 95°C for 10 seconds, and then finally, 55°C for 1 minute. No template controls and no enzyme controls were similarly run on each plate to ensure no contamination was present. Expression levels were calculated for TLR2, NLRP3, and IL6 and were normalized to GAPDH. Normalization and fold change were calculated for each time point and compared to baseline, day 0 samples using the ΔΔCt method as recommended by Bio-Rad.

### Data analysis

All data were plotted using GraphPad Prism (Version 5.04; GraphPad, La Jolla, California) and statistical significance was determined using multiple *t* tests with the Holm-Sidak correction for multiplicity.

## RESULTS

**Figure 1:**
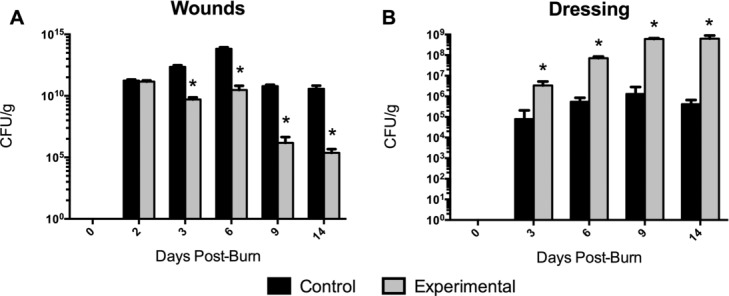
Colony forming units of methicillin-resistant *Staphylococcus aureus* (MRSA) per gram of wound (A) or dressing (B) biopsies quantified at 2, 3, 6, 9, or 14 days after burn injury. Statistically significant differences (*) in MRSA levels between experimental and control dressing samples (B) or the corresponding treated wounds (A) at each time point were assessed using multiple *t* tests with a Holm-Sidak correction for multiple tests (*P* < .05). Data are shown as means with standard deviations.

**Figure 2:**
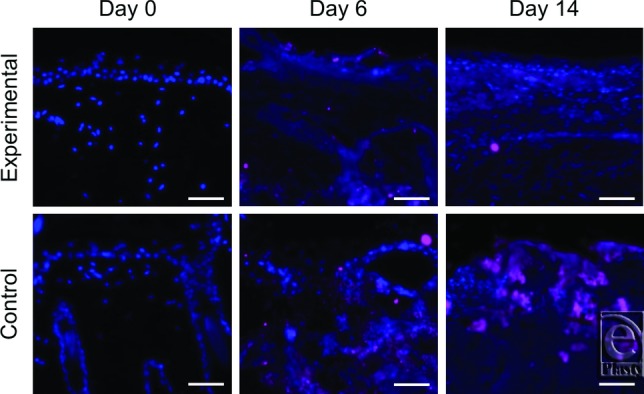
Immunofluorescent staining for the localization of *S aureus*. Representative images from days 6 and 14 show the increased staining of *S aureus* (*pink*) in the control-treated wound biopsies, indicating higher bacterial presence compared to the experimentally treated wounds. DAPI counterstain is blue. Images are taken at 10× magnification and scale bars represent 50 μm.

**Figure 3:**
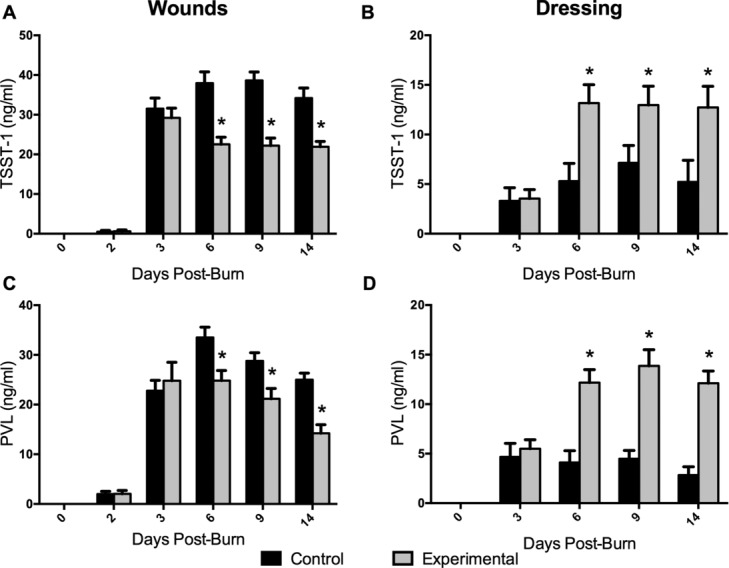
Levels of toxic shock syndrome toxin 1 (A, B) or Panton-Valentine leukocidin (C, D) quantified in wound (A, C) or dressing (B, D) samples at 2, 3, 6, 9, or 14 days after burn injury. Statistically significant (*) differences in toxin levels in control versus experimental dressing (B, D) or corresponding treated wounds (A, C) were determined at each time point using multiple *t* tests with a Holm-Sidak correction for multiple tests (*P* < .05). Data are shown as means with standard deviations.

**Figure 4:**
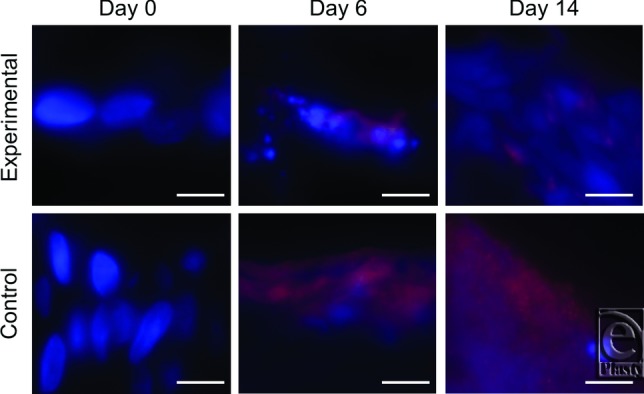
Immunofluorescent staining for the localization of toxic shock syndrome toxin 1 (TSST-1). Representative images from days 6 and 14 show the increased staining of TSST-1 (*red*) in the control dressing-treated wound biopsies, indicating higher toxin concentration compared to the experimentally treated wounds. DAPI counterstain is blue. Images are taken at 100× magnification and scale bars represent 5 μm.

By postburn day 3, significantly less (*P* < .05) MRSA was measured in wounds treated with experimental dressing than in those treated with control dressing. Quantities remained lower in the experimental group on day 6 (*P* < .001) and were further reduced on days 9 (*P* < .001) and 14 (Fig 1A). Correspondingly, more MRSA was quantified in the experimental dressing than in control dressing at all time points (*P* < .05), with levels increasing in the experimental dressing through day 14 (Fig 1B). Wound biopsy images obtained using immunofluorescence show differential *S aureus* staining in wounds treated with experimental versus control dressings (Fig 2). Experimental dressing-treated wounds also contained significantly less TSST-1 (Fig 3A) and PVL (Fig 3C) than controls (*P* < .01) on days 6, 9, and 14, while the experimental dressing itself contained more TSST-1 and PVL on days 6, 9, and 14 than the control dressing (*P* < .001; Figs 3B and 3D). Virulence factor levels were not significantly different between experimental and control groups on day 3 in the wounds or dressings. Low levels of virulence factors were measured in wounds from both groups as early as postburn day 2, only 24 hours after inoculation began (Figs 3A and 3C). Immunofluorescent wound biopsy images show correlative differential TSST-1 staining in wounds treated with experimental versus control dressings (Fig 4).

**Figure 5:**
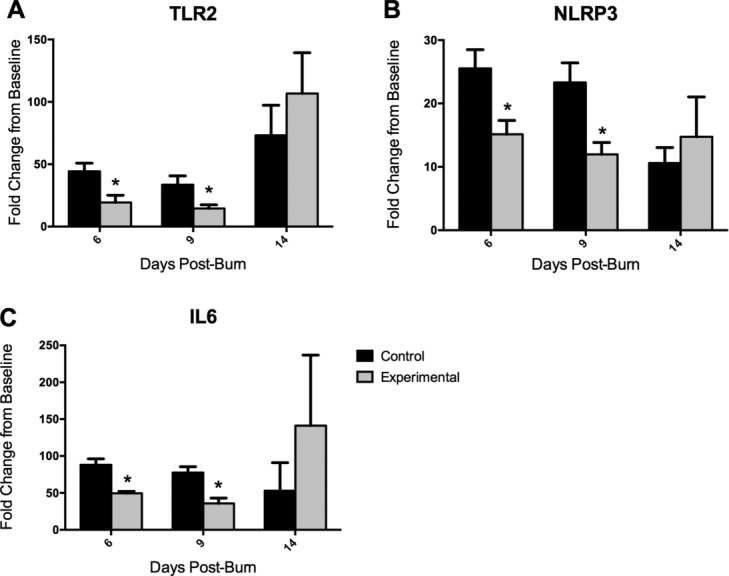
Fold change in mRNA expression of innate immunity-related genes from levels quantified in preinjury, day 0 skin samples. Comparisons between experimental and control dressing-treated wounds in the induction of TLR2 (A), NLRP3 (B), and IL6 (C) at each time point were made using multiple *t* tests with a Holm-Sidak correction for multiple tests (*P* < .05), with statistically significant differences indicated (*). Data are shown as means with standard deviations. GAPDH indicates glyceraldehyde 3-phosphate dehydrogenase; IL6, interleukin 6; NLRP3, NOD-like receptor family, pyrin domain containing 3; TLR2, toll-like receptor 2.

Induction of TLR2, NLRP3, and IL6 was significantly lower in experimental dressing-treated wounds than in controls on days 6 and 9 (*P* < .05; Fig 5). Induction of TLR2, NLRP3, and IL6 appeared to be higher in control dressing-treated wounds than in experimentally treated wounds by day 14, though these differences were not statistically significant (Fig 3).

**Figure 6:**
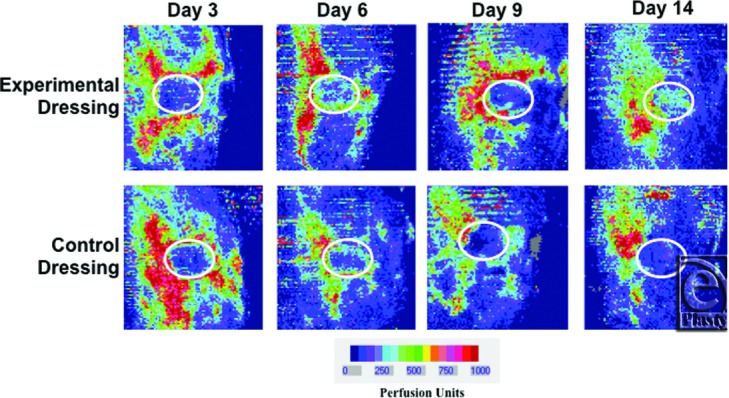
Laser Doppler imaging (LDI) used to quantify perfusion levels in experimentally treated versus control treated wounds. Representative LDI flux images from each time point are shown. White circles represent regions of interest where mean perfusion units were calculated for each wound.

Laser Doppler images taken during the first 3 time points (day 0 postburn, day 1, and day 2) reflect the same levels of perfusion between treated groups (Table 2, before treatment). There were no statistically significant differences in wound perfusion (*P* = .51, *P* = .89, *P* = .61, respectively) during these time points. On day 3, when the dressings had been applied for at least 24 hours, differences in wound perfusion were measurable (Table 2, after treatment and Fig 6) with perfusion in experimental dressing-treated wounds surpassing that measured in control dressing-treated wound by day 14, though none of these differences were statistically significant at any time point.

## DISCUSSION

The experiments described here were designed to assess the in vivo capabilities of the experimental dressing in taking up bacteria and associated virulence factors from infected burn wounds. As the dressing is purported to move large quantities of slough and exudate from wound beds,^22^ it was hypothesized that if this is the case, large amounts of bacteria and proteinaceous virulence factors should also be moved from the wound into the dressing. Furthermore, it would be expected that this reduction or elimination of pathogen from the wound might impact the healing process and host response in some way that is meaningful for more positive outcomes in treated wounds.

The results show that the experimental dressing reduced the quantities of MRSA as well as TSST-1 and PVL to a significant extent in treated wounds, exceeding the reductions provided by the control dressing. Moreover, this reduction and its significant difference from controls was observed within approximately 24 hours of the dressings being applied to the inoculated wounds, potentially demonstrating rapid action that could be clinically valuable in treating invasively infected wounds, as well as preparing wound beds for further treatment. These experiments ran for 14 days after injury, therefore examining the treatment of inoculated wounds with the dressings for 12 days. Over this time course, the experimental dressing showed a continued trend of reducing MRSA levels in the wounds, while a steady increase was observed in the amount contained in the dressing samples through day 14 (Fig 1). Similarly, PVL showed a steady decrease in experimental dressing-treated wounds over the entire time course (Fig 3C). These trends were not precisely replicated in the TSST-1 data, in which the quantities of toxin measured in both the wounds and dressing appeared to level off and remain consistent by approximately day 6 (Figs 3A and 3B). Several considerations may result from these observations.

For one, does the ability of the dressing to continue to take up additional bacteria, but seemingly not toxin, speak to a possible mechanism of action of the hydroconductive dressing? To date, the mechanisms allowing for such significant uptake of exudate and wound debris by this dressing have been reported to be a combination of capillary, hydroconductive, and electrostatic actions,^20^ but no controlled laboratory experiments have been done to confirm this. Perhaps the potential difference in ability to continue to take up these bacteria cells versus a specific virulence factor with a well-defined tertiary structure is based on size or affinity and associated interaction with the dressing. Previous work demonstrated the dressing's capability in accumulating other wound-relevant pathogens and their associated virulence factors, which have different properties,^23^ however this work was done in vitro and over a shorter time course (4 days). Further work should be done to better characterize the potential mechanisms that allow for high levels of effectiveness in accumulating pathogen and virulence factors, as this information could lead to more specific indications for use and enhanced knowledge of which wounds or types of infection the dressing may be most efficacious for.

A second consideration is the dressing's potential saturation point. If the experiment had been extended over a longer time period, would the dressing reach a “saturation” point in which it could not take up any additional pathogen (or virulence factor, or other wound debris)? If in fact a maximum capacity exists for the dressing, further questions would include whether the contaminants taken up by the dressing would eventually then be released back into the wound, or rather, how long they might be retained. By extension, would the dressing be able to reduce the amount of bacteria present in the wound to levels significantly below the threshold of an “invasive infection” (<10^5^ CFUs/g)? While the answers to these questions are likely dependent on the amount of pathogen and virulence factor present when the dressing is applied, in the data presented here, MRSA levels are reduced in the experimental dressing-treated wounds to 2.3 × 10^5^ CFU/g by day 14. Further experiments should be done to evaluate the true capacity of the experimental dressing. This capacity may have clinical implications in terms of frequency of dressing changes, and whether the presence of contaminants in the dressing would, over some amount of time, impact the wound being treated.

In this set of experiments, the experimental dressing was compared to a control foam dressing, which was chosen because of its similarities to the experimental dressing in terms of its physical properties and because it contains no antimicrobials. While the present experiments demonstrate a significantly greater ability of the experimental dressing to remove pathogens from burn wounds compared to this control dressing, the question remains as to whether it may be more effective in eliminating pathogens than a dressing that does contain an antimicrobial. The pathogen elimination by the experimental dressing may very well exceed that of other treatment options including topical antimicrobial agents, and with less cell damage or cytotoxicity; however, future study is needed to examine this. Furthermore, additional studies are needed to determine whether Drawtex as a stand-alone treatment is optimal in cases of burn wound infection and will be studied in future work.

The data resulting from quantitative culture and ELISA methods were in line with what was hypothesized about the capabilities of this dressing based on previous in vitro study. The mRNA transcript changes require additional consideration in terms of the dressing's capabilities, and an extension of the potential for a significant reduction in bacteria and toxin to impact the local host response and healing potential. As is illustrated in Figure 5, experimental dressing-treated wounds show less induction of the innate immune and inflammatory response-related genes on postinjury days 6 and 9 compared to control dressing-treated wounds, though both groups do show some levels of induction (and therefore host response). While the differential induction of TLR2, NLRP3, and IL6 was observable at these earlier time points more proximal to injury, inoculation (day 1), and dressing application (day 2), by day 14, no statistically significant differences were measurable between the 2 groups (Fig 5). This may be indicative of a return to baseline or normalization after an initial host response to pathogen at the transcript level, as NLRP3 (Fig 5B) and IL6 (Fig 5C) control groups both show a trend of decrease from previous time points on day 14. Alternatively, it is likewise observed that the induction in each of these genes in the experimental group becomes higher than that of the control group (though not statistically significant) at day 14, possibly indicating a somewhat delayed or dampened host response. This could be due to the correspondingly lower levels of pathogen in the wounds in the experimental groups early on in the time course.

## CONCLUSION

In summary, the hydroconductive dressing provided a significant reduction in pathogen and virulence factors—exceeding that of a control dressing. Furthermore, as a result of clearance of virulence factors from the wound bed, a requisite reduction in host innate immune response was observed. Additional work should be done to further characterize clinical metrics of healing over a time course in experimental dressing-treated wounds. This should be done in comparison to multiple comparator treatments including those containing antimicrobials.

## Figures and Tables

**Table 1:** Primer sequences used in real-time reverse transcriptase polymerase chain reaction to quantify gene expression for housekeeping gene (GAPDH) and innate immunity-related genes of interest in wound tissue^a^

Gene	Accession number	Forward primer	Reverse primer
GAPDH	NM_017008	5’-GCAAGAGAGAGGCCCTCAG- 3’	5’-TGTGAGGGAGATGCTCAGTG- 3’
TLR2^a^	NM_198769	5’-GCTGTTGCGTTACATCTTGGA-3’	5’-GGCTCCGTATTGTTACCGTTT-3’
NLRP3	NM_001191642	5’-CTGCAGAGCCTACAGTTGGG-3’	5’-ACCCTACACTAAAAGCGCCC-3’
IL6	NM_012589	5’-TTCTCTCCGCAAGAGACTTCC-3’	5’-TCTCCTCTCCGGACTTGTGAA-3’

^a^Primer sequences obtained from previous publications.

Abbreviations: GAPDH, glyceraldehyde 3-phosphate dehydrogenase; IL6, interleukin 6; NLRP3, NOD-like receptor family, pyrin domain containing 3; TLR2, toll-like receptor 2.

**Table 2:** Laser Doppler imaging was used to quantify perfusion levels in experimentally treated versus control treated wounds^a^

	Before treatment			After treatment			
	Day 0 after burn	Day 1 after burn	Day 2 after burn	Day 3 after burn	Day 6 after burn	Day 9 after burn	Day 14 after burn
Experimental	0.96 ± 0.06	0.60 ± 0.02	1.02 ± 0.04	1.07 ± 0.08	0.96 ± 0.11	1.18 ± 0.07	1.48 ± 0.36
Control	0.91 ±0.03	0.59 ± 0.02	0.98 ± 0.02	1.11 ± 0.13	1.34 ± 0.15	0.89 ± 0.27	1.06 ± 0.06

^a^Data are presented as fold change from baseline (Day 0, before burn) ± standard error of mean.
